# Supplementary material for: The Systems Biology Research Tool: evolvable open-source software
Source: BMC Syst Biol. 2008 Jun 29;2:55. doi: 10.1186/1752-0509-2-55 (PMC2446383; doi:10.1186/1752-0509-2-55)
Supplement: Additional file 1 — SBRT Archive. An archive of the current version of the Systems Biology Research Tool. [file 1752-0509-2-55-S1.zip › sbrt-1.4.0/doc/developers_guide/api/sbrt/kernel/util/class-use/MutableProgressMonitor.html]

Uses of Interface sbrt.kernel.util.MutableProgressMonitor


|  |  |  |
| --- | --- | --- |
| **Overview** \| **Package** \| **Class** \| **Use** \| **Tree** \| **Deprecated** \| **Index** \| **Help** | |  |
| PREV   NEXT | **FRAMES**    **NO FRAMES**     **All Classes** |  |


---


## **Uses of Interface sbrt.kernel.util.MutableProgressMonitor**

| Packages that use MutableProgressMonitor | |
| --- | --- |
| **sbrt.kernel.fba.comp** | Provides classes and interfaces for performing Flux Balance Analysis computations. |
| **sbrt.kernel.util** | Provides classes and interfaces for performing utility functions. |

Uses of MutableProgressMonitor in sbrt.kernel.fba.comp

| Methods in sbrt.kernel.fba.comp with parameters of type MutableProgressMonitor | |
| --- | --- |
| `protected  java.util.Map<java.lang.String,java.lang.Double>` | `ReverseRxnFluxIntervalComputer.getFluxMaxima(java.util.Collection<java.lang.String> rxnNames, MutableProgressMonitor progress)`             Computes and returns the maximum fluxes of the reactions with the provided names. |
| `protected  java.util.Map<java.lang.String,java.lang.Double>` | `ReverseRxnFluxIntervalComputer.getFluxMinima(java.util.Collection<java.lang.String> rxnNames, MutableProgressMonitor progress)`             Computes and returns the minimum fluxes of the reactions with the provided names. |
| `SparseDoubleMatrix<?,java.lang.String>` | `SlpExCurrComputerV2.getMatrix(IrrevRxnFluxome fluxome, MutableProgressMonitor pm)`             Returns the extreme current matrix for the provided fluxome. |
| `SparseDoubleMatrix<?,java.lang.String>` | `ExtremeCurrentComputer.getMatrix(IrrevRxnFluxome fluxome, MutableProgressMonitor pm)`             Returns the extreme current matrix for the provided fluxome. |
| `java.util.Map<java.lang.String,java.lang.Double>` | `SimpleFluxIntervalComputer.getMaximumFluxes(java.util.Collection<java.lang.String> rxnNames, MutableProgressMonitor progress)`             Computes and returns the maximum fluxes of the reactions with the provided names. |

Uses of MutableProgressMonitor in sbrt.kernel.util

| Classes in sbrt.kernel.util that implement MutableProgressMonitor | |
| --- | --- |
| `class` | `AbstractProgressMonitor`             This abstract class is a skeleton implementation of `ProgressMonitor`. |
| `class` | `DiscreteProgressMonitor`             This class is used to monitor the progress of a process that occurs in discrete events. |
| `class` | `PercentProgressMonitor`             This class is used to monitor a process whose progress can be measured as a percentage. |

---


|  |  |  |
| --- | --- | --- |
| **Overview** \| **Package** \| **Class** \| **Use** \| **Tree** \| **Deprecated** \| **Index** \| **Help** | |  |
| PREV   NEXT | **FRAMES**    **NO FRAMES**     **All Classes** |  |


---
